# Neurobiology of Vascular Dementia

**DOI:** 10.4061/2011/401604

**Published:** 2011-08-17

**Authors:** Ana-Maria Enciu, Stefan N. Constantinescu, Laurenţiu M. Popescu, Dafin F. Mureşanu, Bogdan O. Popescu

**Affiliations:** ^1^Department of Cellular and Molecular Medicine, School of Medicine, “Carol Davila” University of Medicine and Pharmacy, 8 Eroilor Sanitari, Sector 5, 050474 Bucharest, Romania; ^2^Ludwig Institute for Cancer Research Ltd., de Duve Institute, Université Catholique de Louvain, 1200 Bruxelles, Belgium; ^3^Laboratory of Molecular Medicine, “Victor Babeş” National Institute of Pathology, 99-101 Splaiul Independenţei, Sector 5, 050096 Bucharest, Romania; ^4^Department of Neurology, “Iuliu Hatieganu” University of Medicine and Pharmacy, 8 Victor Babeş, 400023 Cluj-Napoca, Romania; ^5^Department of Neurology, University Hospital Bucharest, “Carol Davila” University of Medicine and Pharmacy, 169 Splaiul Independenţei, Sector 5, 050098 Bucharest, Romania; ^6^International Clinical Research Center, St. Anne's University Hospital Brno, 53 Pekařská, 656 91 Brno, Czech Republic

## Abstract

Vascular dementia is, in its current conceptual form, a distinct type of dementia with a spectrum of specific clinical and pathophysiological features. However, in a very large majority of cases, these alterations occur in an already aged brain, characterized by a milieu of cellular and molecular events common for different neurodegenerative diseases. The cell signaling defects and molecular dyshomeostasis might lead to neuronal malfunction prior to the death of neurons and the alteration of neuronal networks. In the present paper, we explore some of the molecular mechanisms underlying brain malfunction triggered by cerebrovascular disease and risk factors. We suggest that, in the age of genetic investigation and molecular diagnosis, the concept of vascular dementia needs a new approach.

## 1. Vascular Dementia—Historical Considerations

**Figure 1:**
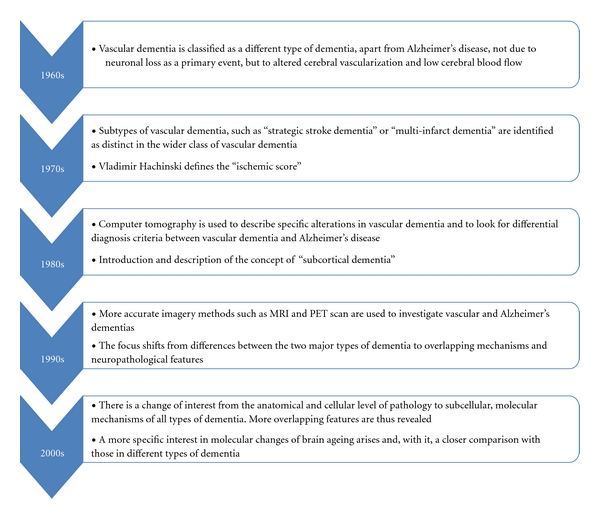
Evolution throughout time of vascular dementia concept.

Just how far back in time should one go when searching data of vascular dementia (VaD)? In 1549, Jason Pratensis published *De Cerebri Morbi, *linking dementia to stroke [1], and in 1658, Johann Jakof Wepfer theorized that a broken brain blood vessel may cause apoplexy (stroke) [2]. The correlation between atherosclerotic disease and dementia was clearly identified only at the beginning of the 20th century by two well-known contributors to the field of neurodegeneration: Alois Alzheimer and Otto Binswanger [3]. The modern era of vascular dementia began in the 1960s, under the leadership of the Newcastle College of Medicine [4]. The concept of VaD was ever since under permanent scrutiny and revision, in light of new clinical, pathological, and imagery data (Figure 1). In the early 1970's, multiple infarct dementia was recognized as a major type of dementia, apart from Alzheimer's disease, characterized not by “neuronal atrophy” but by atherosclerotic burden. In 1975, Vladimir Hachinski defined the “ischemic score,” later used for the clinical diagnostic of vascular dementias [5]. However, the concept of VaD soon became controversial due to an increased discrepancy between the incidence of cognitive disorders and that of the “strategic stroke.” Furthermore, the early prevention of multi-infarct dementia (MID), the aging of the general population, and an arising need to define “normal aging” versus “pathological aging” [6] added to this controversy. The struggle to identify preventable and treatable factors widened the pathogenic spectrum of VaD [7]. Several epidemiological studies reported associations of hypertension, type 2 diabetes, obesity, and inflammation with VAD and, in some cases, AD. These all coincide with those of stroke, which in turn is an established factor for cognitive decline and VAD [8] and underlines furthermore the need for a new classification of dementia types [9]. During the last two decades, there was a switch of exploration from classical pathology to new imaging techniques at the molecular level. Therefore, new pathogenic pathways were identified, which greatly increased the complexity of mechanisms of neuronal loss due to cerebral vascular injury [10].

In each stage of clinical and imaging research, new attempts were made to define VaD as an individual, self-standing class of dementia. Mayer-Gross et al. presented in the late 60s a set a criteria including dementia with focal signs and symptoms consistent with stroke, a fluctuating course, preservation of intellectual powers, and personality until late in the disease. Importantly, definition included vascular risk factors such as hypertension [11].

The “multi-infarct dementia” (MID), first described by Vladimir Hachinski, was characterized by a number of small ischemic strokes that may not result in focal neurologic deficits, but in time, by cumulative damage, would lead to cognitive decline. Later, the Hachinski ischemic scale, used for MID diagnosis, was modified by Loeb and Gandolfo to include CT scan criteria [12].

In the 1990s, as acknowledgment of overlapping features of various types of dementia, VaD criteria included clinical and imaging features of *probable* and *possible* disease. Criteria for *definite* VaD would require histopathological evidence from biopsy or autopsy [13].

Currently, the most widely used criteria for VaD include the *Diagnostic and Statistical Manual of Mental Disorders* (DSM), Alzheimer's Disease Diagnostic and Treatment Centers (ADDTC), *International Statistical Classification of Diseases* (ICD), and National Institute of Neurological Disorders and Stroke—Association Internationale pour la Recherche et l'Enseignement en Neurosciences (NINDS-AIREN) criteria [14].

## 2. The Concept of Brain Ageing

The concept of brain ageing stated at first that cell death might be responsible for the progressive deterioration of different physiological functions. Studies on aged animals [15] from over two decades ago reported neuronal loss with aging, with or without cortical thinning (depending on the type of method used for quantification), but with diminution of the total volume of gray matter. By the end of the 1980s, reports of preserved neuronal number, despite cortical thinning in human brain [16], started to challenge the previous data and were followed by confirmatory studies on animals [17–20]. This controversy was solved by modern imaging investigational methods, starting with computer tomographic analysis in the early 1980's [21] and continuing with recent PET and MRI analyses [22, 23]. These techniques demonstrated that brain atrophy does occur with age in the healthy, nondemented elderly, involving both gray and white matter, but the loss is rather of neuronal connections, not of neurons. Furthermore, quantitation of neurons showed that, despite frontal and medial temporal cortical thinning, the number of neurons is preserved in healthy adults. Freeman et al. reported that, in frontal and temporal neocortical regions, the neuronal count remained relatively constant over a 50-year age range, suggesting that the atrophy is a reflection of the 3D neuronal network loosening rather than perikaryal loss [24]. The prefrontal cortical neurons seem to be particularly vulnerable to ageing, as a decrease in dendritic branching has been reported in neocortex of both rat [25] and human brains [26–28]. By contrast, there is no significant change in dendritic length of hippocampal granule cells, nor a reduction in spine density in the dentate gyrus of aged humans [29] or rats [30].

White matter reduction is also a consistent finding in the aged human brain, possibly as an indicator of defective myelination (although oligodendrocyte number seems to increase). White matter loss is strongly correlated with vascular risk factors, particularly hypertension and stroke [31], two pathologies included in the broad spectrum of VaD risk factors. However, the involvement of white matter abnormalities and the presence of lacunae yielded contradictory results in terms of functional integrity and cognitive impairment [32]. 

At the molecular level, aging is a “decrease in homeostatic reserve” [33] which interferes with neuronal ability to limit and buffer the increase of reactive oxygen species (ROS) production, to sustain a protective response to cytotoxic stimuli or to limit vicious cycles such as inflammatory environments. DNA damage increases with age (some of which is ROS related), somatic mutation in human lymphocytes being nine times more frequent in aged human subjects than in neonates [34], and mitochondrial DNA being even more sensitive than nuclear DNA. Mitochondrial aging brings its share of vulnerability to stress in aged cells, with decreased ATP reserves [35] along with affected cellular calcium removal systems and low buffering capacity [36]. Moreover, one should take into account the fact that, in the brain, these processes affect, at different rates, different cell types that share a homeostatic balance. On the other hand, understanding aging of the nervous tissue, as compared to other tissues, could be a more challenging task due to a more complex regulation, signaling, and intercellular interactions.

## 3. VaD from a Molecular Perspective

The molecular perspective on VaD is rather limited; the general concept of this type of cognitive impairment has derived from clinical and imaging findings and is correlated, at the cellular level, with neuronal death and the sudden interruption of neuronal networks. The main pathological changes leading to different forms of vascular dementia take place in both large (atherosclerosis and thrombosis) and small (lipohyalinosis and fibrosis) cerebral vessels, secondary to common vascular risk factors, such as hypertension, diabetes mellitus, and dyslipidemia. The reduction in cerebral blood flow (CBF) starts early during vascular disease [37] and, therefore, a major vascular event can be preceded by a variable period of chronic hypoxia. As a result, the brain cellular microenvironment might change and adaptive processes may lead to cellular malfunction, rather than cellular death.

### 3.1. Cerebral Blood Flow and Ischemia-Triggered Molecular Events

Normal aging is associated with low cerebral flow and velocity at rest [38] and an attenuation of responsivity to hypoxia, hypercapnia, or blood pressure alterations [39]. These modifications may appear due to either histological alterations of the vessel wall (thickening of basement membrane, loss of pericytes, and an overall reduction in cortical vascular bed) or lower metabolic demand. The same changes in blood flow, but at a higher rate, were documented in subcortical ischemic VaD patients by PET studies, with some groups reporting a preferential decrease in frontal lobe regions [40]. In laboratory rats, chronic hypoxia increases the CBF for several days, after which a decrease towards the baseline is noted, probably due to compensatory mechanisms such as increased hematocrit and decreased metabolic needs [41]. Hypoxia inducible factor-1 (HIF-1) was used by Ritz et al. as a marker of hypoxia in the cortex of young (2 months) and old (9 months) spontaneously hypertensive rats (SHR) and stroke-prone SHR, in their study on hypoxic alterations of nonneuronal populations [42]. Interestingly, the increase in HIF1*α* was documented only in aged animals, along with an imbalance between microvessels and astrocytes at the level of the neurovascular unit. In hypoxic conditions, HIF-1*α* is upregulated, dimerizes with HIF-1*β* (the constitutively expressed subunit of HIF-1), translocates into the nucleus, and binds to hypoxia-responsive elements (HREs) of target genes, such as vascular endothelial growth factor (VEGF), glucose transporter-1 (GLUT1), lactate dehydrogenase (LDH), erythropoietin (Epo), and nitric oxide synthase (NOS).

### 3.2. Inflammatory Cytokines, Adhesion Molecules, and Endothelial Malfunction

Endothelial malfunction is considered to be a first step in the development of atherosclerosis, and may be objectified by overexpression of inflammatory cytokines and adhesion molecules, leading to monocyte recruitment in the nascent atherosclerotic plaque and overproduction of reactive oxygen species (ROS), as a sign of mitochondrial, peroxisomal, and lysosomal alteration.

Measurements of plasma markers in VaD patients showed increased levels of proinflammatory cytokines (IL1, IL6, TNF*α*) as well as anti-inflammatory cytokines (IL-10) [43]. IL-6 and TNF*α* levels increase with aging in animals and humans, and IL-6 transgenic mice also show progressive proliferative cerebellar angiopathy and blood-barrier (BBB) breakdown. These events indicate the endothelium as one of the main targets of proinflammatory cytokine IL-6 [44]. The same transgenic strains indicated for the first time a causative relationship between local production of IL-6 in the brain and the age-related decline in learning and cognitive function, demonstrating dendritic vacuolization, stripping of dendritic spines, decreased synaptic density, and loss of GABA-producing neurons in the hippocampus. In association with neurodegenerative changes, a diffuse nonproliferative gliosis with marked activation of astrocytes and microglia was identified in GFAP-IL6 mice [45]. Furthermore, studies in transgenic mice overexpressing TNF and/or its receptors (p55 and p75^NTR^) demonstrated that IL-6 is a potent microglial activator and, depending on the receptor it activates, (i) an endothelial activator (via p75^NTR^), leading to increased expression of adhesion molecules, BBB disruption, and CNS leukocyte infiltration or (ii) a demyelinating agent and oligodendrocyte apoptosis inducer via p55 [46]. According to Batti and O'Connor, although TNF*α* has no effect on synaptic transmission or long-term potentation (LTP) under basal conditions, it severely impairs the recovery of postsynaptic transmission after hypoxic exposure [47]. They also showed that the TNF*α* effect is p38/MAPK mediated, a signaling pathway involved as well in hypoxic neuronal death in the CA1 region of the hippocampus. But, in addition to its neurotoxic nature, TNF*α* may also exert neuroprotective effects [48, 49] in selected signaling contexts.

Suggested to be another marker of chronic inflammation [50], E-selectin is an endothelial adhesion molecule, that is involved in weak linking of circulating leukocytes. Its expression is upregulated by IL-1 and TNF*α*. Elevated levels of E-selectin have been previously linked to experimental and clinical brain ischemia [51], and high levels of soluble selectin (sE-selectin) have been correlated with severe cerebrovascular disease [50]. Generating immune tolerance against E selectin by repeated low-dose mucosal administration in lab rats had a protective effect against hemorrhagic strokes in HRS rats and against VCI development in Wistar rats, as shown by Wakita et al. [52].

### 3.3. Oxidative Stress

The impact of ROS on cognitive function is elegantly demonstrated by studies of superoxide dismutase (SOD) isoenzyme transgenic mice. Overexpression of mitochondrial SOD has a neuroprotective role against drug-induced neurotoxicity, overexpression of cytoplasmic SOD improves age-related impairments in LTP, and overexpression of extracellular SOD is correlated with better spatial memory in laboratory rats [53]. Following cerebral ischemia, the production of free radicals was increased in aged rats and human endothelial cells, mainly by overproduction in the monocyte/macrophage system, especially when stimulated by inflammatory mediators [54].

### 3.4. Effect of VaD Molecular Alterations on Neuronal and Glial Populations

Hypoxia is associated with increased expression of all NO synthase isoforms, including neuronal (nNOS), astrocyte and microglia-inducible isoform (iNOS), and endothelial isoform (eNOS) [55], which are involved in neuronal death through inhibition of mitochondrial respiration and NMDA/Ca^2+^-induced exotoxicity [56, 57]. Brain cells are particularly sensitive to ROS aggression due to their high content of polyunsaturated fatty acids, which constitute a substrate for lipid peroxidation. Exposure of brain cells to oxidative stress increases the accumulation of cholesterol in cell membranes [58], leading to decreased fluidity and impaired transmembrane transport.

Hypoxia also upregulates the expression of BDNF—a neurotrophic factor with important roles in neuroplasticity and hippocampus-related learning. This might serve as a protective mechanism against a paucity of hippocampal BDNF mRNA and BDNF plasma levels at older ages [59]. BDNF is further reduced by vascular risk factors such as hypertension and poor glucose metabolism [60]. However, hypoxic upregulation of BDNF is not accompanied by upregulation of its high-affinity receptor Trk-B, but of its low-affinity receptor p75^NTR^, a TNF superfamily receptor. The p75^NTR^ expression is upregulated by hypoxic conditions and is correlated with an increase in caspase-3 activation in cortical and hippocampal neurons, leading to apoptosis [61]. The upregulation of p75^NTR^ is linked to NOS stimulation and to Ca-mediated regulation of expression, suggesting a complex transformation of the pattern of molecular expression in chronic ischemia and VaD.

## 4. Mixed versus Pure Dementia

 “Mixed” dementia is, by the very definition of Vladimir Hachinski himself, “Alzheimer's disease and cerebral infarcts contributing to the dementia” [6], but other coexisting pathologies are also common in dementia such as Parkinson disease (in about 20% of patients with AD) and dementia with Lewy bodies (up to 50%) [62].

 Many data suggest that “pure” vascular dementia is rare and is the exception, rather than the rule [63–65]. Vasculopathy as a trigger of AD neuropathological features has been proposed repeatedly before [66–68], and it is very likely that a patient with late-onset AD may already have a vascular burden and shares with VaD vascular risk factors. Moreover, Zhang et al. demonstrated that the low-oxygen dependent increase in HIF1*α* expression was accompanied by an increase of BACE1 protein levels and a secondary increase in A*β* production [69]. These data suggest that restoration of normal oxygen levels to hypoxic tissues, for example, by the use of small molecules that lower the affinity of oxygen for hemoglobin, could be an interesting issue for research [70, 71]. 

Activation of inflammation is a consistent finding in AD, as shown in cell culture models [72, 73], animal models [74, 75], and postmortem studies on AD brains [76–78]. 

Inflammation was related to the onset of cognitive decline and also correlated with disease progression by measurements of serum TNF*α* and the TNF*α*/IL1-*β* ratio. Patients with AD show elevated levels of TGF-*β* that are correlated with low expression of TGF-R in the affected brain areas, especially around cerebral vessels with CAA [45]. Furthermore, inflammation is associated with ROS production, and oxidative stress has a dual relationship with A*β* peptide: (i) it favors the aggregation of A*β* into a fibrillar form and (ii) it mediates the toxic effect of A*β* on neuronal cells, as shown by the protective effect of antioxidants and free radical scavengers [79]. In turn, some A*β* peptides (such as the 25–35 form) have an intrinsic lipoperoxidizing effect, as established on neocortex-derived synaptosomes [80]. Oxidative stress is demonstrated by the increased amount of 4-hydroxynonenal (HNE), which was shown to interfere with plasmalemmal ATPases and transporters, including Ca^2+^ shifters, further increasing metabolic imbalance in AD. 

Downstream A*β* production and accumulation results in secondary endothelial malfunction through: (i) amyloid angiopathy; (ii) NOS inhibition [50]; (iii) atherogenesis correlated with endothelial activation and overexpression of inflammatory cytokines and adhesion molecules, even before A*β* deposition [81]; (iv) lipid peroxidation in the frontal cortex in AD brains [82]; (v) BBB alteration [83].

**Figure 2:**
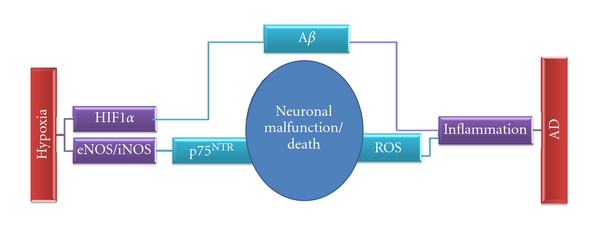
Some mechanisms converging towards neuronal malfunction in two major types of dementia.

To conclude, there is an overlap of events between chronic hypoxia and AD on several levels, such as hypoxic-triggered cellular pathways, inflammatory environment, growth factor signalling, and calcium homeostasis (Figure 2). Thus, from the molecular level perspective, the diagnostic criteria for neurodegenerative diseases have become ill defined or insufficient and there is a true need for redefinition.

## 5. Overlapping of Normal Aging and Neurodegenerative Diseases at Cellular and Molecular Level

Normal aging and various types of neurodegeneration share common molecular events (Table 1), such as alteration of cerebral blood flow, neuroinflammatory environment, and endothelial malfunction.

Aging favors the production of proinflammatory cytokines, mostly through microglial and astrocytic activation [54]. Aging has also been associated, at the cellular level, with increased production of reactive oxygen species (ROS) [109]. Oxidative alteration of enzymes and the subsequent loss of enzymatic activity is a trait of the aging brain, particularly, in the anterior frontal lobe [49]. Oxidative stress leads to the accumulation of free cholesterol [79], along with ceramides, lipid peroxides, and derived aldehydes (such as HNE), that covalently bind to membrane proteins, altering their functions.

Oxidative stress is involved as well in the disruption of Ca^2+^ homeostasis, an effect studied especially in neurons, where Ca^2+^ is a vital mediator of neuronal signaling. It appears that, in aged neurons, several Ca^2+^ homeostatic systems are affected [33] and there is impairment in the maintenance of a nontoxic Ca^2+^ overload [120]. 

Although it seems that levels of nNOS and eNOS do not change with age, still there is an increase in NOS activity in aged rat cortex. These two NOS isoforms are Ca^2+^ induced, which correlates with the above-mentioned impairment of aged cells to deal with Ca^2+^ overload. Furthermore, consistent with the Ca^2+^-independent nature of iNOS, there are several reports underlining its absence in the normal aged cortex of lab rats [15, 60, 105].

## 6. Conclusions

Instead of considering VaD a pure result of neuronal death and the interruption of neuronal networks that support cognitive function, we hypothesize that early brain malfunction is induced by vascular risk factors and chronic hypoxia. A reduction of CBF and a series of molecular events precede the major ischemic events in vascular cognitive impairment. Based on these subtle changes, intervention at early stages could prevent the full-blown development of dementia, which might represent a “point of no return” for the neurovascular units and neuronal networks with few chances for effective treatment.

## Figures and Tables

**Table 1:** Comparison between normal aging and neurodegenerative diseases from a molecular perspective.

Parameter	Normal aging	Vascular dementia	Alzheimer's disease	Other neurodegenerative disorders
CBF	Diminished with lower velocity, but with preserved dynamic adaptability [84]	Diminished in parietal and frontal lobes, some authors reported also a decrement in superior temporal gyri, thalami, anterior cingulate gyri [85]	Diminished only in parietal cortices and later in advanced disease in frontal lobes [86]	Diminished in preoccipital and occipital regions in PD [87] and LBD [88]

VEGF -A	Low basal levels produced by astrocytes [89]	Upregulation of VEGF and VEGF R2 in astrocytes [90]	Low serum levels and decreased secretion by peripheral immune cells [91]	FTLD—associated with VEGF gene promoter polymorphism in selected populations [92]

Inflammatory cytokines				
IL-6	Increased mRNA compared to young subjects [93]	High blood levels, associated with high CRP may be associated with high risk [94]	Positive immunoreactivity in amyloid plaques and increased concentration in AD brain, compared to age-matched subjects [95]	Increased in cerebral and cerebellar cortex of Huntington patients [96]
TNF*α*	Increased basal levels in aged laboratory animals with week induction injury response [97]	Modulates neuronal cell loss in cerebral ischemia [98]	Increased expression in AD brain, along with TNF-R1 [99]	Increased in plasma [100], CSF of PD patients and in PD brains, especially in areas with greatest loss of dopaminergic neurons [101]
TGF*β*1	Detected at low levels in CSF and produced in CNS at low levels by neuronal cells [102]	Increased in CNS and CSF after stroke [103]	Increased in areas with amyloid burden [104]	CAA—directly related to amyloid vascular deposition [105]

Adhesion molecules	sVCAM increased [106]	sVCAM increased in atherosclerotic disease [107]; sE-selectin increased in severe cerebrovascular disease [108]	sVCAM elevated in late onset AD [50]	sVCAM increased in Down Syndrome [100]

ROS	Increased accumulation with aging [109]	Increased in ischemia animal models and stroke patients [110]	Increased: A*β*-related ROS generation and MAOS [111]	Increased in PD *in vitro* models [112] and animal models [113]

Lipid metabolism	Accumulation of ceramides and free cholesterol in cerebral cortex [114]	Hypercholesterolemia is a known risk factor for VaD	Increased levels of cholesterol, and activation of cholesterol biosynthesis pathway [115]	PD dementia does not correlate with apoE polymorphism or lipid profile [116]
GLUT 1	Altered structure and function of GLUT-1 [117]	Downregulated in prolonged hypoxia [118]	Low expression in AD hippocampus and double transgenic APP/PS1 animal model Learning increases expression in mouse brain [119]	Insufficiently investigated in neurodegeneration, but involved in “Glut-1 deficiency syndrome”— a treatment-resistant form of epilepsy [120]

BDNF	Decreased mRNA in human plasma and hippocampus [121]	Increased expression following hypoxic stress in cell cultures [122, 123] and lab animals [123]	Decreased expression in hippocampus temporal and frontal cortex [124]	Reduced BDNF expression in the caudate and putamen in HD patients [96] Reduced mRNA BDNF expression [125] and protein [126] in striatal neurons in PD patients

Calcium	Reduced homeostatic reserve [33]	Involved in ischemia-induced excitotoxicity [127]	A*β* disrupts Ca homeostasis in cortical neuronal cell cultures [117]	Excitotoxicity and excessive Ca^2+^-mediated nitric oxide production are believed to contribute to the death of dopaminergic neurons in PD [118]; Huntingtin transgenic mice express mitochondrial Ca overload upon glutamate stimulation [119]

MAOS: membrane-associated oxidative stress VDCC: voltage dependent calcium channels, FTLD: frontotemporal lobar dementia, LBD: Lewy body dementia, and HD: Huntington disease.

## Abbreviations

A*β*: Amyloid beta peptide
p75^NTR^: Low affinity receptor for tumor necrosis factor *α*
HIF 1*α*: Hypoxia Inducible Factor 1*α*
ROS: Reactive oxygen species
eNOS: Endothelial nitric oxide synthase
iNOS: Inducible nitric oxide synthase.
